# Family caregiving in the community up to 8-years after onset of dementia

**DOI:** 10.1186/s12877-020-01613-9

**Published:** 2020-06-19

**Authors:** Eric Jutkowitz, Joseph E. Gaugler, Amal N. Trivedi, Lauren L. Mitchell, Pedro Gozalo

**Affiliations:** 1 Department of Health Services, Policy & Practice, Brown University School of Public Health, Box G-S121-6, 121 S. Main Street, 6th Floor, Providence, RI 02912 USA; 2 Providence Veterans Affairs (VA) Medical Center, Center of Innovation in Long Term Services and Supports, Providence, Rhode Island 02908 USA; 3 Division of Health Policy and Management, School of Public Health, University of Minnesota, Minneapolis, MN 55455 USA; 4 Center for Care Delivery & Outcomes Research, Minneapolis VA Healthcare System, One Veterans Drive, Minneapolis, MN 55417 USA

**Keywords:** Community based long-term care, Alzheimer’s disease and related dementias, Longitudinal, Health services, Public health

## Abstract

**Background:**

Persons with Alzheimer’s disease and related dementias (ADRD) receive care from family/friends, but how care changes from the onset of dementia remains less understood.

**Methods:**

We used the Health and Retirement Study (2002–2012) to identify community-dwelling individuals predicted to have incident ADRD. We investigated the amount of caregiving received for activities of daily living in the 8-years after disease onset.

**Results:**

At incidence (*n* = 1158), persons with ADRD received 151 h (SD = 231) of caregiving a month, 25 (SD = 26) caregiving days a month and had 1.3 (SD = 1.4) caregivers a month. By 8-years post incidence, 187 (16%) individuals transitioned to a nursing home and 662 (57%) died in the community. Community-dwelling persons with ADRD at 8-years post incidence (*n* = 30) received 283 h (SD = 257) of caregiving, 38 (SD = 24) caregiving days, and had 2.2 (SD = 1.3) caregivers.

**Conclusions:**

Community-dwelling persons with ADRD receive a substantial amount of caregiving over the first 8-years after disease onset.

## Background

Persons with Alzheimer’s disease and related dementias (ADRD) require a significant amount of long-term care [1–3], and because of this they incur $56,290 more in care per year than persons without ADRD [4]. A majority of ADRD costs are incurred by families through the value of caregiving and out-of-pocket expenditures [2–7].

Most ADRD care is provided in the community by family (e.g. spouse and adult children) and friend caregivers [8]. Caregivers support a variety of tasks including managing comorbidities and assisting with functional limitations (e.g., grocery shopping and dressing), and they provide general supervision of the person with ADRD [9–11]. Compared to persons without ADRD that receive caregiving, persons with ADRD receive substantially more hours and intensity of care [9, 10]. Providing more hours of ADRD caregiving is associated with caregiver depression, physical illness, employment complications, and family conflict [12, 13]. While persons without ADRD receive most care from a spouse, persons with ADRD are more likely to receive care from adult children. ADRD caregiving can be challenging, time consuming and stressful, but it can also be rewarding [14–16].

Although most persons with ADRD desire to remain in the community [17–20], demographic trends threaten the current ADRD caregiving paradigm [8, 17, 19, 21, 22]. Currently, there are 7 caregivers for every older adult ≥80 years of age, but this ratio is projected to decrease by 2050 to less than 3 to 1 [23, 24]. To successfully enable individuals with ADRD to remain in the community requires high quality affordable community-based care and having available family caregiver(s) [8, 17, 19]. With the predicted decline in availability of caregivers and the greater number of older adults with impending chronic care needs such as ADRD, there will be greater pressure on fewer individuals within families to provide caregiving.

To help families, providers and policy makers plan for ADRD caregiving needs, it is vital to understand community-based ADRD caregiving patterns over the disease trajectory, during which persons with ADRD experience cognitive and functional decline and responsive behaviors [5, 25]. To date, most studies on ADRD caregiving are cross-sectional; even longitudinal studies tend to include samples of caregivers that have provided care for limited durations [25–29]. In general, longitudinal studies have found that managing behavioral responses, poorer physical functioning, comorbidity, and marital status are associated with a greater risk of institutionalization and receiving more caregiving [28–31]. The short duration of studies represents a major limitation in our understanding of ADRD caregiving given its intensity and the variation in clinical interventions and services needed over the course of the disease. Understanding longitudinal patterns of caregiving (starting with disease onset) can inform both the type and timing of different long-term care services and supports that can best meet the changing needs of persons with ADRD and their caregivers (e.g., promoting respite programs, such as adult day services, much sooner following ADRD onset) [32].

The Health and Retirement Study (HRS) is a nationally representative longitudinal survey of US adults ≥51 years. It is an ideal dataset to evaluate ADRD caregiving patterns over the course of the disease as it contains a validated approach for identifying persons with ADRD and it collects detailed information on the caregiving received by respondents [9, 10, 25]. Using data from the HRS, we investigated the caregiving received by persons with ADRD while they remained in the community from up to 8-years post incidence. We expect the amount of caregiving received by persons with ADRD will increase over time. Finally, as noted above prior empirical evaluations have identified positive associations with individual (e.g., functional ability) and family-level measures (e.g., marital status) and time spent caregiving [28–31]. Therefore, in exploratory analyses we investigated the association between similar individual/family level predictors and the amount of caregiving received over the course of the disease.

## Methods

### Data

We used data from 2002 to 2014 of the publicly available HRS (Core HRS files and RAND 2014 V2 files) [33]. We used data from 2002 onward because they represent the first year HRS changed their approach to identify caregiving support. HRS initially samples from community-dwelling adults, but once a respondent enters the study they are followed until death. Periodically, HRS adds new cohorts to the study so as to maintain a nationally representative sample. Nearly all baseline interviews are conducted face-to-face. Follow up interviews are conducted approximately every 2 years, where half the sample participates face-to-face and the other half participates by telephone. During HRS interviews demographic, economic, family, and medical information are collected on the core respondent and their spouse. Data are also collected on the care respondents receive. If a respondent is unable to participate in a survey, then a proxy respondent is identified.

This secondary analysis of HRS data was approved by the Institutional Review Board of Brown University under protocol 3#1810002244.

### ADRD incident cohort

We used the validated Langa-Weir algorithm to identify community-dwelling HRS respondents predicted to have ADRD. The Langa-Weir ADRD algorithm combines the immediate and delayed recall test, serial 7 subtraction test, and backward count from 20 test to generate a wave specific cognitive score to predict if a respondent has normal cognition, cognitive impairment but not dementia, or dementia (i.e., ADRD) [34–36]. For respondents with a proxy, the Langa-Weir algorithm predicts respondent cognitive status based on the proxy’s assessment of the respondent’s memory and functional limitations and if the respondent was unable to complete the survey due to cognitive limitations. The Langa-Weir algorithm was validated against ADRD diagnoses from the Aging, Demographics, and Memory Study and was found to correctly classify 76% of self-respondents and 84% of respondents with a proxy [35].

To identify community-dwelling ADRD incident cases, we identified the first HRS interview a respondent had ADRD between 2002 and 2012 according to the Langa-Weir algorithm. We did not use 2014 data to identify incident cases, as these individuals would automatically be censored following 2014 (the last year of data available in the RAND HRS 2014 V2 files). However, we did follow incident cases identified from 2002 to 2012 up to 2014. Finally, we excluded HRS respondents that had ADRD in one wave but in subsequent waves did not based on the Langa-Weir algorithm.

In addition to the inclusion criteria described above, we excluded persons with ADRD that had data linkage issues across core HRS files and/or that had inconsistent nursing home entry or death dates. We also excluded individuals that had missing data on model covariates (described below). We limited our analysis to up to 8 years post incidence due to the small sample size of individuals with ADRD still in the community at 8 years (*n* = 30). We also determined if each respondent transitioned to a nursing home, died while in the community, dropped out of the HRS/did not participate in an HRS interview, or remained in the sample/was censored due to the study design.

### Measures

#### Caregiving received

HRS respondents report if they received help from any individuals (i.e., caregivers) when performing instrumental (IADL; preparing hot meals, shopping for groceries, making telephone calls, and taking medications) and/or basic activities of daily living (ADL; getting across a room, dressing, bathing, eating, getting in/out of bed, and toileting). For each identified caregiver, the respondent reports their relationship to the caregiver and the number of hours and days in a month that caregiver provided assistance. We categorized caregivers as a spouse, adult child, other relative, nonrelative, or paid caregiver. An individual was classified as being a paid caregiver if they were employed by an organization or were a nonrelative that was paid to provide assistance. For each respondent and HRS interview, we calculated the total monthly hours of caregiving received, number of caregiving days in a month caregiving was received (i.e., sum of days of care provided by all caregivers), and number of monthly caregivers. We assumed caregivers could provide at most 16 h of care per day [37]. Our measure of the number of caregiving days could exceed 30 days (e.g., spouse and adult child each provide 20 days of care which is equivalent to 40 care days).

#### Individual and family characteristics

We obtained the respondent’s age, gender, race (white, African American, other [American Indian, Alaskan Native, Asian, and Pacific Islander]), years of education, number of chronic conditions (0–8; high blood pressure or hypertension, diabetes or high blood sugar, cancer [except skin cancer], lung disease except asthma or emphysema, heart attack/coronary heart disease/angina/congestive heart failure/or other heart problems, stroke, psychiatric problems, or arthritis), whether they were enrolled in Medicaid, whether they had long-term care insurance, and if they had a proxy respondent. The RAND HRS reports whether a respondent has any difficulties (0 = no difficulty; 1 = any difficulty) performing IADLs (preparing hot meals, shopping for groceries, making telephone calls, taking medication; 0–4) and ADLs (getting across a room, dressing, bathing, eating, getting in/out of bed, and toileting; 0–6) [38]. Respondents are asked to only report difficulties that are expected to last more than 3 months. We determined the total number of functional limitations (0–10) a respondent had in each HRS wave by summing the binary indicators across the measures of IADLs/ADLs. Finally, we obtained information on the respondent’s family characteristics including their net worth, marital status, number of sons, number of daughters, number of married children, number of living siblings, number of grandchildren, and number of great grandchildren.

### Statistical analysis

We evaluated the characteristics of the person with ADRD, their family, and the amount of caregiving received in the community approximately every 2 years (mean time between interviews was 1.95 years (SD = 0.35)) from incidence up to 8-years post incidence. At each time post incidence, we also evaluated the characteristics of those who transitioned to a nursing home or died in the community.

In preliminary analyses, we observed that the measures of caregiving received were associated with transitions to nursing homes and mortality. Therefore, to estimate the association between individual and family-level characteristics of the person with ADRD and receiving care in the community we estimated a series of joint mixed effects and survival models [39]. We estimated separate joint models for each measure of caregiving outcome (hours of caregiving, days of caregiving, and number of caregivers). The joint modeling framework consists of two parts that share parameters and the models are estimated with a joint likelihood function. In the first part, we estimated a random intercept and slope mixed effects model where the outcome represents the measure of caregiving received (hours of caregiving received, caregiving days, and number of caregivers). In the second part, we estimated a Weibull survival model in which the outcome represented nursing home placement or mortality in the community (whichever occurred first). Withdrawal from the HRS, missing an HRS interview, and being alive and in the community in 2014 or at 8-years post incidence were treated as censoring events. We modeled the association between the mixed effects and Weibull survival model using the current value parameterization method [39].

## Results

**Fig. 1:**
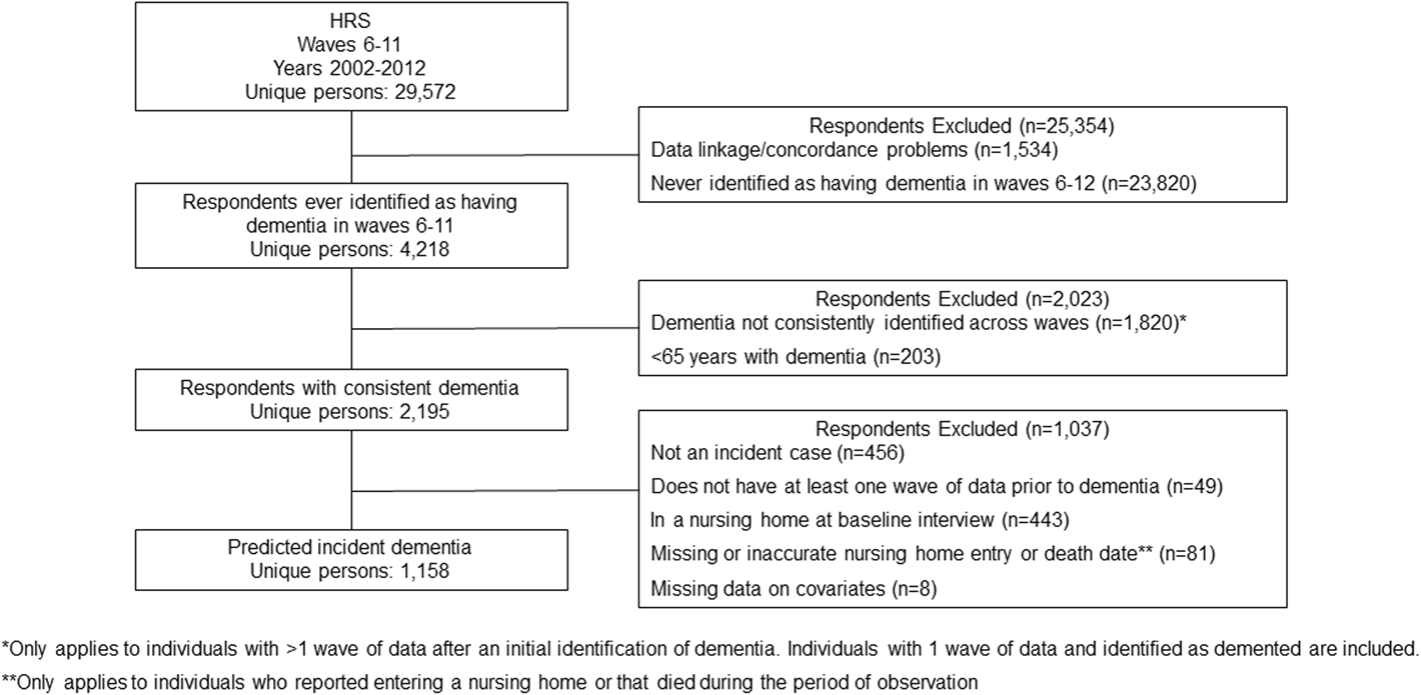
Derivation of Sample

Of the 29,572 persons in HRS (2002–2012), 1158 met our definition of incident ADRD (Fig. 1). At incidence, mean age of persons with ADRD was 81.4 (SD = 7.5) years, most were female (60%), white (80%), and had 3.2 (SD = 3.2) functional limitations (Table 1).

**Table 1:** Sample Characteristics

	Incidence *N* = 1158	2-years Post Incidence *N* = 508	4-years Post Incidence *N* = 183	6-years Post Incidence *N* = 76	8-years Post Incidence *N* = 30
Age, mean (SD), y	81.4 (7.5)	82.8 (7)	83 (7)	83.6 (7.1)	84.7 (7.3)
Educational, mean (SD), y	10.9 (3.6)	10.7 (3.7)	10.1 (3.7)	9.4 (3.9)	9 (3.8)
Female, n (%)	694 (59.93)	329 (64.76)	124 (67.76)	52 (68.42)	15 (50)
Race, n (%)					
White	928 (80.14)	403 (79.33)	130 (71.04)	46 (60.53)	17 (56.67)
African American	184 (15.89)	86 (16.93)	45 (24.59)	27 (35.53)	12 (40)
Other^a^	46 (3.97)	19 (3.74)	8 (4.37)	3 (3.95)	1 (3.33)
Number of functional limitations (0–10), mean (SD)	3.2 (3.2)	3.8 (3.2)	4.7 (3.3)	5.6 (3.4)	4.9 (3.8)
Number of chronic conditions (0–8), mean (SD)	3 (1.6)	3 (1.6)	3.1 (1.6)	3.4 (1.4)	3.9 (1.6)
Medicaid, n (%)					
Yes	177 (15.3)	85 (16.7)	39 (21.3)	21 (27.6)	9 (30.0)
No	940 (81.2)	407 (80.1)	134 (73.2)	52 (68.4)	21 (70.0)
Unknown	41 (3.5)	16 (3.2)	10 (5.5)	3 (4.0)	0 (0.00)
Long-term care insurance, n (%)					
Yes	98 (8.5)	57 (11.2)	15 (8.2)	9 (11.8)	2 (6.7)
No	1014 (87.6)	436 (85.8)	158 (86.3)	63 (82.9)	27 (90.0)
Unknown	46 (4.0)	15 (3.0)	10 (5.5)	4 (5.3)	1 (3.3)
Proxy respondent, n (%)	420 (36.3)	232 (45.7)	99 (54.1)	49 (64.5)	16 (53.3)
Net worth, mean (SD), $	377,534 (1,585,096)	320,275 (809,095)	255,612 (490,421)	295,121 (758,520)	195,305 (410,057)
Marital status, n (%)					
Married/partnered	529 (45.68)	215 (42.32)	73 (39.89)	31 (40.79)	12 (40.0)
Separated /divorced	92 (7.94)	37 (7.28)	15 (8.2)	5 (6.58)	3 (10.0)
Widowed	513 (44.3)	248 (48.82)	91 (49.73)	39 (51.32)	14 (46.7)
Never married	24 (2.07)	8 (1.57)	4 (2.19)	1 (1.32)	1 (3.3)
Number of sons, mean (SD)	1.7 (1.5)	1.8 (1.5)	1.9 (1.5)	2.1 (1.5)	2.3 (1.6)
Number of daughters, mean (SD)	1.7 (1.6)	1.7 (1.5)	1.9 (1.4)	2 (1.4)	1.9 (1.6)
Number of married children, mean (SD)	2.3 (1.8)	2.3 (1.8)	2.3 (1.8)	2.6 (1.7)	2.4 (1.5)
Number of living siblings, mean (SD)	2 (2.2)	2.1 (2.3)	2.3 (2.4)	2.6 (2.8)	2.4 (2.3)
Number of grandchildren, mean (SD)	6.65 (6.1)	6.52 (5.7)	7.63 (6.4)	8.30 (6.4)	7.23 (4.3)
Number of great grandchildren, mean (SD)	0.23 (0.6)	0.27 (0.7)	0.26 (0.7)	0.25 (0.5)	0.23 (0.5)
No caregiver, n (%)	393 (33.9)	121 (24.8)	35 (19.1)	8 (10.5)	3 (10.0)
Spouse, mean (SD)	0.27 (0.4)	0.28 (0.5)	0.30 (0.5)	0.32 (0.5)	0.23 (0.4)
Adult child, mean (SD)	0.53 (0.9)	0.71 (1.0)	0.84 (0.9)	0.95 (1.0)	0.93 (0.8)
Other relative, mean (SD)	0.24 (0.6)	0.31 (0.7)	0.37 (0.7)	0.47 (0.7)	0.60 (0.8)
Nonrelative, mean (SD)	0.06 (0.3)	0.05 (0.3)	0.02 (0.2)	0.01 (0.1)	0.10 (0.3)
Paid caregivers, mean (SD)	0.21 (0.5)	0.31 (0.7)	0.30 (0.6)	0.48 (0.8)	0.37 (0.6)

^a^Other race includes American Indian, Alaskan Native, Asian, and Pacific Islander

Over time, the number of persons with ADRD that remained in the community decreased. By 2-years post incidence, 10% (*n* = 112) of the incident cohort had transitioned to a nursing home, 38% (*n* = 439) had died, and 8% (*n* = 99) were lost to follow up (eTable 1 and eTable 2). At 8-years post incidence, 3% (*n* = 30) of the incidence cohort remained in the community. At all times post incidence, mortality and nursing home admission represented the primary reasons for attrition from the community.

### Caregiver relationship, hours of caregiving received, days of caregiving received, and number of caregivers

**Fig. 2:**
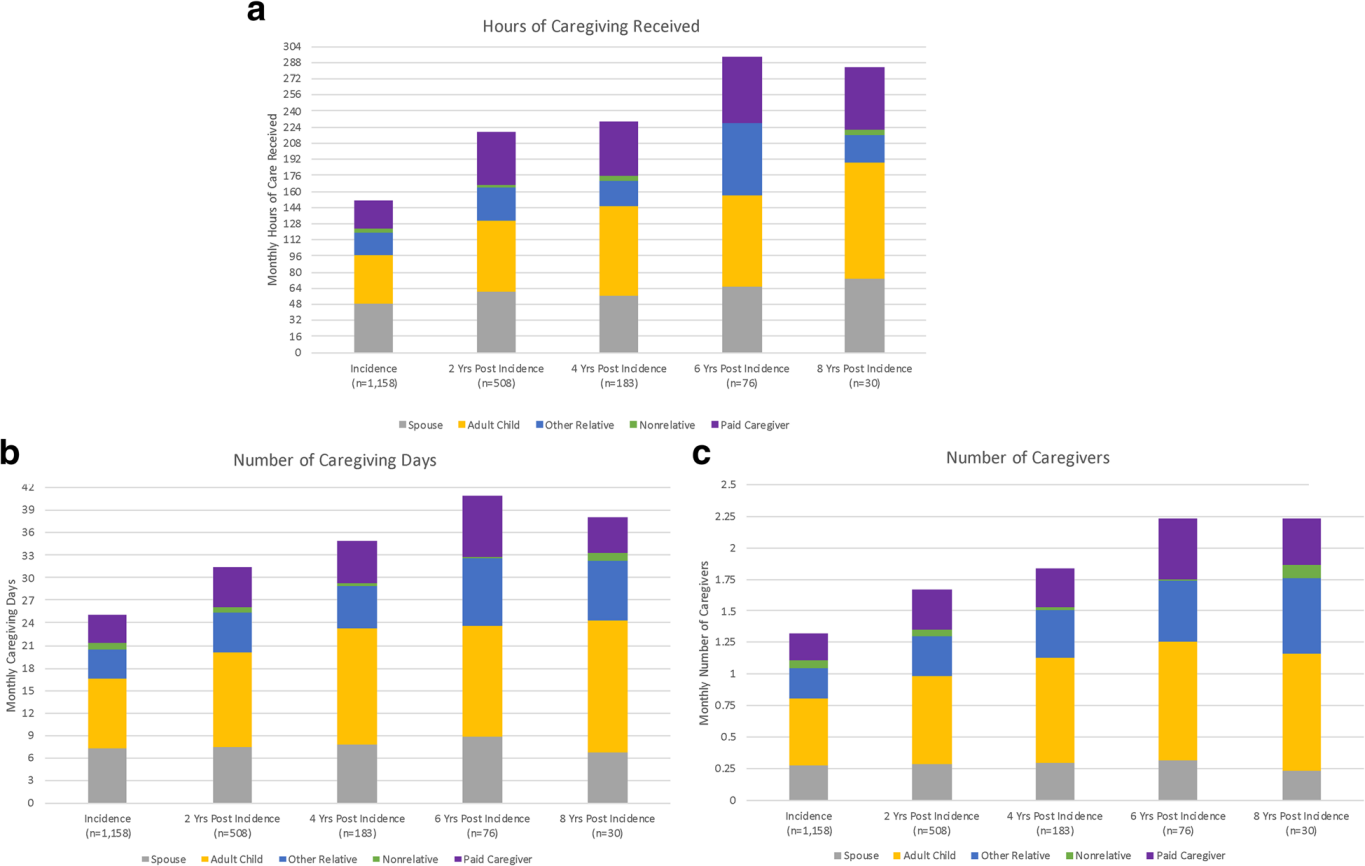
Hours of Care Received, Number of Caregiving Days, and Number of Caregivers

At incidence, 66% (*n* = 765) of respondents reported receiving caregiving (48% [*n* = 738] for those who self-reported vs, 98% [*n* = 420] for proxy-reported; *p* < 0.001 for difference). Over time the proportion of all respondents reporting receiving any caregiving increased. At incidence, persons with ADRD received a mean of 151 (SD = 49) hours of caregiving a month (Fig. 2a) and most of this care was provided by a spouse (33% of total hours) and adult children (32% of total hours). Among those that remained in the community, the hours of caregiving received increased over time. By 8-years post incidence, persons with ADRD that remained in the community received a mean of 283 (SD = 257) hours of caregiving. At all times post incidence, adult children provided the greatest proportion of caregiving hours. Across all years, respondents with a proxy reported receiving on average more hours of caregiving (319.1 h [SD = 168.7]) than respondents who self-reported (86.7 h [SD = 276.9]; *p* < 0.001 for difference with proxy reports) during the HRS interviews (eFigure 1a).

Corresponding with increases in the hours of caregiving received were increases in caregiving days per month (Fig. 2b) and number of caregivers per month (Fig. 2c). At incidence, persons with ADRD received on average 25 (SD = 26) caregiving days, and by 8-years post incidence those that remained in the community received 38 caregiving days (SD = 24). Similarly, at incidence persons with ADRD had a mean of 1.3 (SD = 1.4) caregivers, but at 8-years post incidence they had on average 2.2 (SD = 1.3) caregivers which was due to an increase in the number of adult children, other relatives and paid caregivers. Compared to respondents who self-reported the amount of caregiving received, respondents with a proxy reported receiving more days (44.7 [SD = 25.02] for proxy-reported vs, 16.7 [SD = 22.4] for those who self-reported; *p* < 0.001 for difference) (eFigure 1b) and having more caregivers (2.3 [SD = 1.4] for proxy-reported vs, 0.9 [SD = 1.2] for those who self-reported; *p* < 0.001 for difference) (eFigure 1c).

All else equal, each additional functional limitation was associated with receiving 39 (95%CI: 36.1, 42.6) more monthly hours of caregiving and having a proxy respondent was associated with receiving 71 (95%CI: 50.5, 91.8) more hours of caregiving (eTable 3). Furthermore, the presence of a great grandchild was associated with receiving 18 (95%CI: 4.9, 30.7) more monthly hours of caregiving, those who were classified as “other” race relative to whites received 54 (95%CI: 6.1, 102.7) more monthly hours of caregiving, and every $10,000 in net worth was associated with receiving 0.07 (95%CI: 0.01, 0.1) additional hours of caregiving. Similar associations where observed for effects on days of caregiving and number of caregivers. Factors associated with leaving the community (i.e., nursing home admission or mortality), obtained from the joint model of hours caregiving, included age at incidence (HR 1.02 95%CI: 1.01, 1.03), number of functional limitations (HR 1.06, 95%CI: 1.01, 1.1), and number of chronic conditions (HR 1.09, 95%CI: 1.05, 1.2) (eTable 3). These factors were consistent in the joint model evaluating days of care and number of caregivers.

## Discussion

We extend prior research on community-based ADRD caregiving by examining caregiving from disease onset up to 8-years post incidence [9, 10]. At all times, spouses and adult children were the largest providers of community-based ADRD caregiving. Over time, the proportion of caregiving provided by adult children relative to spouses increased, which was due to the needs of the care recipient [5], the fact that individuals on average had multiple children that could potentially provide care but only one spouse, and in some cases may also represent spousal caregivers’ functional decline or mortality [40]. At disease onset, persons with ADRD received 151 h of caregiving, which is equivalent to 4.8 daily hours over the entire month. At 8-years post incidence, those that remained in the community (3% of the original cohort) received 283 h of monthly caregiving or on average over 9 h of care every day of the month.

The number of persons living with ADRD is expected to increase, but the number of family caregivers is expected to decrease [1, 24]. Concurrently, the United States has a fragmented long-term care system with limited public financing and limited availability of affordable paid caregiving/formal long-term care [8]. Most long-term care is financed by individuals and it is not uncommon for people with ADRD to spend down most of their savings on long-term care [41, 42]. Without an increase in the number of family caregivers or affordable long-term care, many persons with ADRD face the potential of living alone in the community or moving to a residential care facility and spending down to Medicaid. This reality is further highlighted by the relatively few individuals in our sample with long-term care insurance. While long-term care insurance can help alleviate challenges associated with caregiving, these plans can be costly and may be unaffordable for many families. The low uptake of long-term care insurance, particularly among lower income families, has been documented in other older adult populations [43].

Initially some individuals with ADRD may be able to live independently; however, over time they will require assistance [6]. In our study, ~ 34% (*n* = 393; 2% [*n* = 420] for proxy-reported vs, 52% [*n* = 738] for those who self-reported) of persons with ADRD at incidence did not report receiving caregiving, but at 8-years post onset only 10% (*n* = 3) of persons with ADRD and still in the community reported no caregiving support for IADLs/ADLs. Although some persons with ADRD reported receiving no caregiving, in reality they may still be receiving care. For several reasons we may be underestimating the amount of caregiving received by persons with ADRD. First, the framing of the caregiving questions in HRS and other national surveys results in variation in the identification of caregivers [44]. Second, HRS respondents report the care received only for specific IADLs/ADLs, but caregivers provide assistance for many other domains (e.g., behavioral issues, socioemotional support, and medical/nursing tasks) [11]. Finally, persons with cognitive impairment may not be able to recall the individuals that provide assistance or the hours of care these individuals provide. We observed stark differences in the amount of caregiving received by a respondent’s proxy status. Having a proxy respondent may mean an individual has greater limitations, but proxy respondents may also have better recall than a person with cognitive impairment.

Overall, we found that paid caregiving was used less frequently than unpaid caregiving; however, ADRD respondents with proxies reported receiving more caregiving from paid individuals compared to their counterparts without a proxy. In general, receiving more hours of family caregiving is associated with receiving less paid caregiving/formal help [45]. Yet, whether receiving more caregiving days and having more caregivers influences the person with ADRD’s outcomes is unknown. It is clear that persons with ADRD receive caregiving from multiple individuals, and that groups of caregivers likely play important roles in the person with ADRD’s health and in providing valuable respite for the primary caregiver.

Consistent with prior literature, we found that the number of functional limitations was an important predictor of hours of caregiving received, days of caregiving received, and of the number of caregivers [46]. The presence of great grandchildren and net worth were also associated with receiving caregiving. Additional research is needed to untangle the mechanisms through which great grandchildren and net worth operate on caregiving. These measures may capture the ability of wealthier people to stay in the community or they may capture the availability of a large network of potential caregivers.

We used the validated Langa-Weir algorithm to identify persons with ADRD, but the algorithm is subject to error. In validation studies the algorithm correctly classified 76–84% of persons with ADRD [34]. Although the predictive characteristics of the Langa-Weir algorithm are similar to other classification methods [47], we may not be capturing individuals earlier in the disease trajectory and with potentially lower caregiving needs. Relatedly, the mortality observed in our sample was elevated when compared to longitudinal clinical studies that use a rigorous clinical diagnosis to identify persons with ADRD [48]. HRS has limitations in the approaches available to identify persons with ADRD; yet, unlike clinical studies the HRS sample is more representative and contains more detailed information on the amount of caregiving received.

Our study has several additional limitations. Due to the design of the HRS we do not report the financial, emotional (both positive and negative), burden or physical impact of caregiving on family caregivers. Furthermore, there is a lag (~ 2-years) between HRS interviews, which means we may not observe important fluctuations in caregiving patterns. Our analysis starts at ADRD incidence; however, it is likely that trajectories of caregiving are initiated in the months and years preceding ADRD diagnoses [24]. Finally, our sample is primarily female (60%), white (80%), and had an average age of 81.4 years at onset. Results should be interpreted and extrapolated with these characteristics in mind.

## Conclusions

Community-dwelling persons with ADRD receive a substantial amount of caregiving from disease onset up to 8-years post onset. Over time, the amount of caregiving received by persons with ADRD increases and is substantial. Long-term care policy must fully support the changing roles of family caregivers that help persons with ADRD live in the community. Examples of policies which may benefit persons with ADRD and their families include comprehensive national long-term care financing, and the dissemination and implementation of effective caregiver support programs [49]. Finally, additional research is needed to understand the effect of caregiving arrangements on persons with ADRD’s health care utilization and time in the community.

## Supplementary information

**Additional file 1: eTable 1.** Attrition by year post incidence. **eTable 2.** Cumulative Attrition Table, n (%). **eTable 3.** Person with ADRD Characteristics Associated with Hours of Care Received, Caregiving Days, and Number of Caregivers. **eFigure 1.**

## Data Availability

We have deposited specific information regarding Stata code used in the analyses in an electronic repository as a guide for investigators who obtain the requisite data use agreement for the relevant data sources and want to replicate our study (https://osf.io/5fqae/?view_only=a55f6e9b4abf4d3f8932bd7cd91d389e).

## Abbreviation

ADRD: Alzheimer’s disease and related dementias
HRS: Health and Retirement Study
IADL: Instrumental activities of daily living
ADL: Activities of daily living
