# Supplementary material for: Family caregiving in the community up to 8-years after onset of dementia
Source: BMC Geriatr. 2020 Jun 19;20:216. doi: 10.1186/s12877-020-01613-9 (PMC7304188; doi:10.1186/s12877-020-01613-9)

Supplemental Online Data

Family Caregiving in the Community up to 8-Years after Onset of Dementia

eTable 1. Attrition by year post incidence.

|  | Incidence | 2-Yrs Post Incidence | 4-Yrs Post Incidence | 6-Yrs Post Incidence | 8-Yrs Post Incidence |
| --- | --- | --- | --- | --- | --- |
| Alive and participated in interview and in community (analysis sample) | 1,158 | 508 | 183 | 76 | 30 |
| Transitioned to nursing^a^ | 0 | 112 | 52 | 18 | 5 |
| Lost to follow up^b^ | 0 | 99 | 15 | 4 | 1 |
| Censored | 0 | 0 | 101 | 39 | 20 |
| Died prior to Wave | 0 | 439 | 157 | 46 | 20 |

^a^ We assume that once a person enters a nursing home, they stay in the nursing home for the remainder of the study.

^b^ We assume that once a person withdraws from the HRS or misses an HRS interview they are lost to follow up.

eTable 2. Cumulative Attrition Table, n (%)

|  | Incidence | 2-Yrs Post Incidence | 4-Yrs Post Incidence | 6-Yrs Post Incidence | 8-Yrs Post Incidence |
| --- | --- | --- | --- | --- | --- |
|  | Starting Sample N =1,158 | | | | |
| Alive and participated in interview and in community (analysis sample) | 1,158 (100) | 508 (44) | 183 (16) | 76 (7) | 30 (3) |
| Transitioned to nursing home^a^ | 0 (0) | 112 (10) | 164 (14) | 182 (16) | 187 (16) |
| Lost to follow up^b^ | 0 (0) | 99 (9) | 114 (10) | 118 (10) | 119 (10) |
| Censored | 0 (0) | 0 (0) | 101 (9) | 140 (12) | 160 (14) |
| Died | 0 (0) | 439 (38) | 596 (51) | 642 (55) | 662 (57) |

^a^ We assume that once a person enters a nursing home they stay in the nursing home for the remainder of the study.

^b^ We assume that once a person withdraws from the HRS or misses an HRS interview they are lost to follow up.

eTable 3. Person with ADRD Characteristics Associated with Hours of Care Received, Caregiving Days, and Number of Caregivers

|  | Hours of caregiving received in a month | | Number of caregiving days received in a month | | Number of caregivers in a month | |
| --- | --- | --- | --- | --- | --- | --- |
|  | Linear mixed effects model | Weibull model^a^ | Linear mixed effects model | Weibull model^a^ | Linear mixed effects model | Weibull model^a^ |
|  | Coefficient  (95% CI) | Hazard Ratio (95% CI) | Coefficient (95% CI) | Hazard Ratio (95% CI) | Coefficient (95% CI) | Hazard Ratio (95% CI) |
| Intercept | -132.2  (-260.43, -3.96) | 1  (1, 1) | -23.89  (-37.19, -10.59) | 0.04  (0.01, 0.11) | -1.71  (-2.44, -0.98) | 0.04  (0.01, 0.11) |
| Years since ADRD onset | 9.81  (2.95, 16.66) |  | 0.65  (0.08, 1.22) |  | 0.05  (0.01, 0.08) |  |
| Age at incidence | 1.18  (-0.2, 2.56) | **1.02**  **(1.01, 1.03)** | 0.26  (0.12, 0.4) | 1.02  (1.01, 1.03) | 0.01  (0.01, 0.02) | 1.02  (1.01, 1.03) |
| Years of education | 2.96  (0.22, 5.71) | 1.01  (0.99, 1.03) | 0.32  (0.03, 0.6) | 1.01  (0.99, 1.03) | 0.02  (0.01, 0.04) | 1.01  (0.99, 1.03) |
| Female | -13.13  (-33.81, 7.55) | 0.73  (0.62, 0.85) | 0.05  (-2.1, 2.19) | 0.73  (0.62, 0.84) | 0.09  (-0.03, 0.21) | 0.73  (0.62, 0.84) |
| Race (ref = White) |  |  |  |  |  |  |
| African American | 22.75  (-4.45, 49.95) | 0.8  (0.64, 0.99) | 3.51  (0.72, 6.3) | 0.79  (0.63, 0.99) | 0.24  (0.09, 0.4) | 0.79  (0.64, 0.99) |
| Other | **54.38**  **(6.1, 102.65)** | 0.81  (0.55, 1.17) | 1.62  (-3.36, 6.6) | 0.81  (0.56, 1.17) | 0.11  (-0.17, 0.38) | 0.82  (0.57, 1.19) |
| Number of functional limitations | **39.37**  **(36.13, 42.62)** | **1.06**  **(1.02, 1.12)** | **4.43**  **(4.1, 4.77)** | 1.06  (1, 1.14) | **0.24**  **(0.22, 0.26)** | 1.07  (1.02, 1.13) |
| Number of chronic conditions | 3.51  (-2.6, 9.62) | **1.09**  **(1.05, 1.15)** | 0.75  (0.12, 1.38) | 1.09  (1.04, 1.15) | 0.04  (0, 0.07) | 1.09  (1.05, 1.15) |
| Medicaid (ref = no) |  |  |  |  |  |  |
| Yes | -1.03  (-25.81, 23.76) | 0.89  (0.73, 1.09) | 0.27  (-2.31, 2.85) | 0.89  (0.72, 1.09) | -0.09  (-0.23, 0.05) | 0.9  (0.73, 1.11) |
| Unknown | -37.01  (-82.73, 8.72) | 0.94  (0.61, 1.43) | -2.16  (-6.93, 2.6) | 0.93  (0.61, 1.42) | 0.18  (-0.08, 0.44) | 0.91  (0.6, 1.4) |
| Long-term care insurance (ref = no) |  |  |  |  |  |  |
| Yes | -15.91  (-46.01, 14.19) | 0.89  (0.69, 1.13) | -0.23  (-3.36, 2.89) | 0.88  (0.69, 1.13) | -0.08  (-0.25, 0.09) | 0.88  (0.69, 1.12) |
| Unknown | -4.08  (-48.7, 40.55) | 1.2  (0.8, 1.77) | -3.42  (-8.04, 1.21) | 1.23  (0.83, 1.82) | -0.23  (-0.48, 0.03) | 1.22  (0.82, 1.8) |
| Proxy respondent | **71.12**  **(50.47, 91.77)** | 1.02  (0.86, 1.22) | **9.81**  **(7.66, 11.95)** | 1.02  (0.83, 1.25) | **0.4**  **(0.28, 0.52)** | 1.04  (0.87, 1.23) |
| Net worth, units of $10,000 | **0.07**  **(0.01, 0.13)** | 1  (1, 1) | **0**  **(0, 0.01)** | 1  (1, 1) | **0**  **(0, 0)** | 1  (1, 1) |
| Marital status (ref = married/partnered) |  |  |  |  |  |  |
| Separated /divorced | -5.18  (-40.19, 29.83) | 1.14  (0.85, 1.52) | 0.76  (-2.89, 4.41) | 1.13  (0.84, 1.51) | 0.25  (0.05, 0.45) | 1.12  (0.83, 1.51) |
| Widowed | 8.33  (-13.68, 30.34) | 1.03  (0.87, 1.22) | 1.5  (-0.78, 3.78) | 1.03  (0.86, 1.22) | 0.24  (0.12, 0.37) | 1.02  (0.85, 1.22) |
| Never married | 4.94  (-60.01, 69.89) | 1.13  (0.68, 1.88) | 1.13  (-5.65, 7.9) | 1.12  (0.67, 1.86) | 0.08  (-0.28, 0.45) | 1.11  (0.66, 1.84) |
| Number of sons | -1.13  (-10.26, 8.01) | 0.99  (0.92, 1.06) | 0.16  (-0.78, 1.1) | 0.98  (0.91, 1.05) | -0.02  (-0.07, 0.03) | 0.99  (0.92, 1.06) |
| Number of daughters | 5.27  (-3.71, 14.26) | 0.97  (0.9, 1.04) | 1.5  (0.57, 2.44) | 0.96  (0.9, 1.04) | 0.04  (-0.01, 0.09) | 0.97  (0.9, 1.04) |
| Number of married children | -0.18  (-9.23, 8.88) | 1  (0.93, 1.08) | 0.31  (-0.62, 1.25) | 1  (0.93, 1.08) | 0.04  (-0.01, 0.09) | 1  (0.93, 1.08) |
| Number of living siblings | 1.5  (-2.86, 5.86) | 0.98  (0.95, 1.02) | 0.37  (-0.08, 0.82) | 0.98  (0.95, 1.02) | 0.02  (-0.01, 0.04) | 0.98  (0.95, 1.02) |
| Number of grandchildren | -0.63  (-3.06, 1.81) | 1  (0.98, 1.02) | -0.28  (-0.53, -0.02) | 1  (0.98, 1.02) | 0.01  (-0.01, 0.02) | 1  (0.98, 1.02) |
| Number of great grandchildren | **17.81**  **(4.89, 30.72)** | 0.96  (0.86, 1.07) | 4.12  (2.77, 5.46) | 0.95  (0.84, 1.08) | 0.15  (0.07, 0.22) | 0.97  (0.86, 1.08) |
| Hours caregiving |  | 1  (1, 1) |  |  |  |  |
| Caregiving days |  |  |  | 1  (1, 1) |  |  |
| Number of caregivers |  |  |  |  |  | 1  (0.9, 1.2) |

Notes: Values discussed in the main text are bolded.

^a^ Failure is modeled as nursing home placement or mortality in the community (whichever occurred first).

eFigure 1


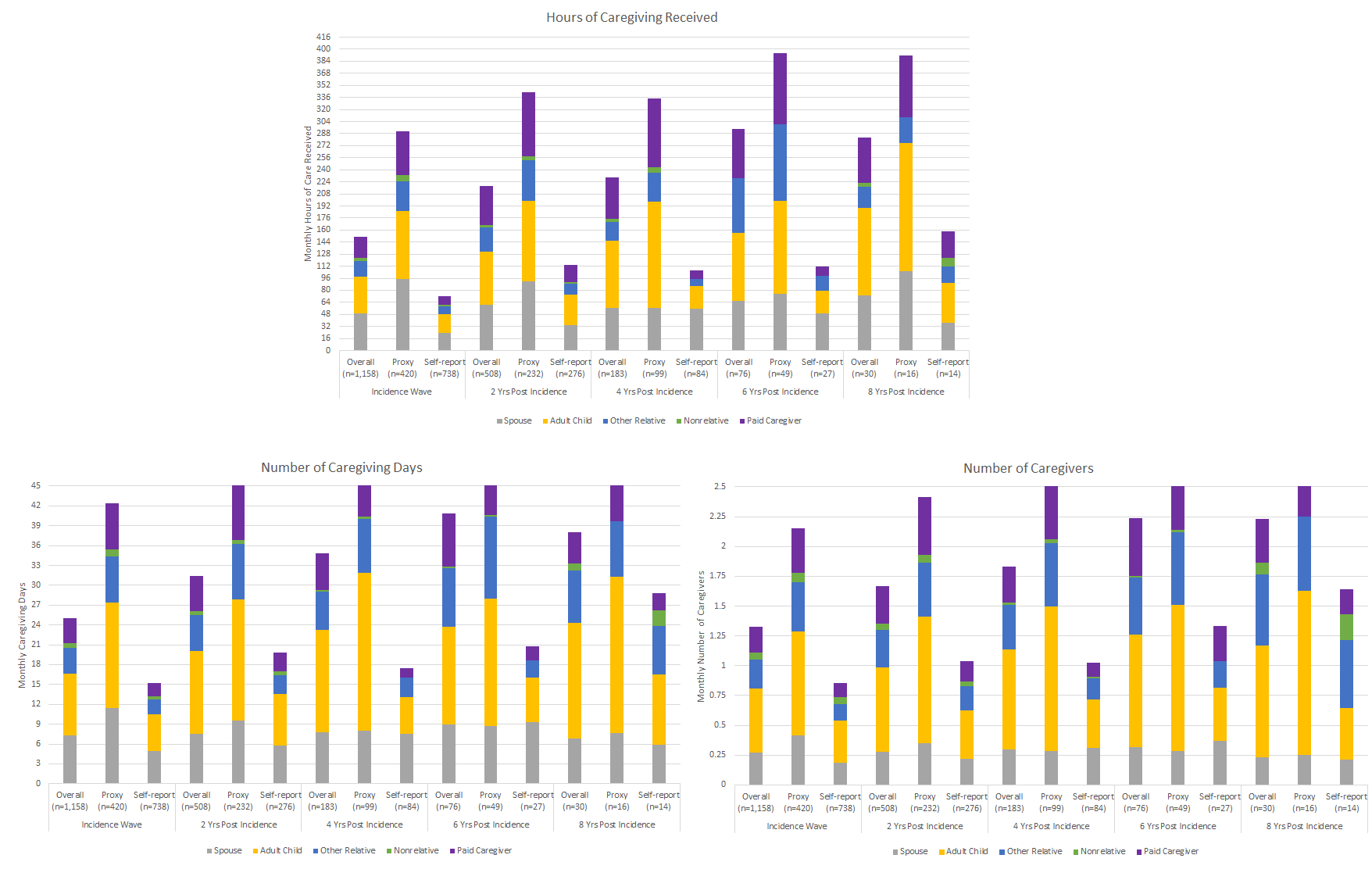

Supplement: Supplementary file 1 — Additional file 1: eTable 1. Attrition by year post incidence. eTable 2. Cumulative Attrition Table, n (%). eTable 3. Person with ADRD Characteristics Associated with Hours of Care Received, Caregiving Days, and Number of Caregivers. eFigure 1. [file 12877_2020_1613_MOESM1_ESM.docx]
